# Dihydropyrimidine Dehydrogenase Genotype–Phenotype Correlations in Plasma and Peripheral Blood Mononuclear Cells

**DOI:** 10.1002/cpt.70511

**Published:** 2026-10-06

**Authors:** Brianna M. Bembenek, Seid Hamzic, Kelly J. Bouchonville, Carlo R. Largiadèr, Steven M. Offer

**Affiliations:** ^1^ Mayo Clinic Graduate School of Biomedical Sciences Mayo Clinic Rochester Minnesota USA; ^2^ Department of Pathology, Carver College of Medicine University of Iowa Iowa City Iowa USA; ^3^ Department of Clinical Chemistry, Inselspital, Bern University Hospital University of Bern Bern Switzerland; ^4^ Graduate School for Cellular and Biomedical Sciences University of Bern Bern Switzerland

## Abstract

The chemotherapeutic 5‐fluorouracil (5‐FU) is commonly used to treat solid tumors, especially colorectal cancer. Deleterious variants in *DPYD,* which encodes dihydropyrimidine dehydrogenase (DPD), are strongly associated with severe and potentially lethal 5‐FU toxicity. Phenotypic assays for DPD function have been proposed as alternative or complementary approaches to identify DPD deficiencies with the rationale that they may capture unrecognized causes. These assays include the direct measurement of DPD activity in peripheral blood mononuclear cell (PBMC) lysates and measuring plasma metabolites uracil (U), dihydrouracil (UH_2_), and the dihydrouracil/uracil (UH_2_:U) ratio. We evaluated correlations between PBMC DPD activity and plasma metabolite measures in matched samples from 203 patients with available *DPYD* exome sequencing data. We also compared the ability of phenotypic measures to identify carriers of deleterious *DPYD* variants. In addition to established high‐risk variants, we identified three rare *DPYD* variants predicted to be deleterious: c.274C>G, c.966C>A, and c.1435G>A. Collectively, these variants were associated with impaired DPD function with effect sizes comparable to well‐studied toxicity‐associated variants. At the population level and within carriers of specific genotypes, weak but significant correlations were observed between PBMC DPD activity and either UH_2_:U ratio or U level. These findings suggest that although phenotypic assays can detect severe DPD deficiency, high variability limits their utility as standalone diagnostic tools. Integration with genetic testing may provide a more robust pretreatment risk assessment strategy.

**Study Highlights**

**WHAT IS THE CURRENT KNOWLEDGE ON THE TOPIC?**

Severe adverse reactions to cancer therapies that include 5‐fluorouracil (5‐FU) occur frequently in patients with reduced function of the dihydropyrimidine dehydrogenase (DPD) enzyme. Genotype‐guided dosing has been shown to effectively reduce toxicity rates in DPD‐deficient patients while maintaining similar drug exposures and overall survival. However, the low sensitivity of genetic tests for 5‐FU toxicity makes phenotypic biomarkers attractive potential alternatives with the potential to capture DPD deficiencies with unknown genetic causes. These tests include blood plasma dihydrouracil (UH_2_) to uracil (U) ratio and measurement of DPD activity in peripheral blood mononuclear cells (PBMCs) as a proxy for liver DPD function, with the latter often considered the “gold standard.”

**WHAT QUESTION DID THIS STUDY ADDRESS?**

In this study, we assessed correlations between plasma U levels, UH_2_:U ratios, PBMC DPD activity, and genotyping from full *DPYD* exome sequencing in a cohort of 203 individuals to identify trade‐offs between tests for identifying individuals that would benefit 5‐FU dose adjustment. We also sought to identify the extent to which phenotypic measures could identify carriers of clinically actionable deleterious *DPYD* variants.

**WHAT DOES THIS STUDY ADD TO OUR KNOWLEDGE?**

Using matched U, UH_2_:U, and PBMC DPD activity measurements, we observed limited correlation between metabolite levels/ratios and PBMC DPD activity. Phenotypic measures showed varying capacities to identify carriers of known deleterious *DPYD* alleles, with plasma metabolite tests showing an advantage over PBMC DPD activity.

**HOW MIGHT THIS CHANGE CLINICAL PHARMACOLOGY OR TRANSLATIONAL SCIENCE?**

These findings suggest that although phenotypic assays can detect severe DPD deficiency, their high variability limits their utility at the individual patient level. A more robust pretreatment risk assessment strategy would include genetic testing combined with phenotypic measures.

Fluoropyrimidines, including 5‐fluorouracil (5‐FU) and its oral prodrug capecitabine, are widely used in the treatment of various solid malignancies. However, severe and life‐threatening adverse events (e.g., CTCAE grade ≥ 3 toxicities) occur in up to one third of patients treated with standard doses.^1^, ^2^, ^3^ 5‐FU is rapidly metabolized to inactive metabolites by dihydropyrimidine dehydrogenase (DPD, encoded by *DPYD*) in the liver. Deleterious *DPYD* variants reduce DPD activity and substantially increase the risk of fluoropyrimidine‐associated toxicity.^4^ Genotype‐guided dose adjustment has shown strong promise for mitigating 5‐FU toxicity without affecting overall survival.^5^, ^6^ However, *DPYD* variants explain only a minor fraction of toxicity cases,^7^, ^8^ and actionable clinical guidance is limited to a subset of well‐characterized alleles. These risk variants include c.1905+1G>A (rs3918290; a.k.a. *2A), c.1679T>G (rs55886062; a.k.a. *13 or p.I560S), c.2846A>T (a.k.a. rs67376798 or p.D949V), and c.1129‐5923C>G (a.k.a. rs75017182; the causal variant linked to the “HapB3” haplotype^9^), as well as additional variants with lower levels of supporting evidence.^10^, ^11^

Phenotypic measures of DPD function have the potential to identify individuals at elevated risk of severe toxicity who have DPD deficiencies that are not due to known genetic causes. While the liver is the major site of 5‐FU catabolism,^12^ DPD activity in peripheral blood mononuclear cells (PBMCs) has been used as a less invasive proxy,^13^, ^14^ and is often considered the “gold standard” approach.^15^ A moderate correlation between DPD activity in PBMCs and liver tissues has been noted in a small study of 27 patients (*r* = 0.56, *R* ^2^ = 0.31)^16^ In addition, approaches for assessing systemic DPD function that are more amenable to routine clinical care, including measurement of plasma uracil (U) and/or the ratio of dihydrouracil (UH_2_) to U (i.e., UH_2_:U ratio).^15^, ^17^ However, metabolite levels have also been suggested to be highly variable and highly standardized pre‐analytical processes necessary when monitoring plasma U.^18^, ^19^

The goals of this study were to assess correlations between PBMC DPD activity and plasma metabolite measures of DPD function within individuals, to evaluate the ability of phenotypic measures to identify carriers of deleterious *DPYD* variants, and to characterize genotype–phenotype relationships to identify additional variants associated with reduced DPD function.

## MATERIALS AND METHODS

### Study population

The population used for this study has been described previously.^9^ Inclusion criteria consisted of adults (≥ 18 years) without prior chemotherapy exposure with self‐declared race as “white.” Study subjects were donors to the Mayo Clinic Biobank and are not intentionally enriched for any disease state including cancer.^20^ Subjects were screened by genotyping for four *DPYD* variants associated with increased risk of 5‐FU‐related toxicity (c.1905+1G>A, c.1679T>G, c.2846A>T, and c.1129‐5923C>G), as described.^9^ For each carrier of c.1129‐5923C>G, two age‐ and sex‐matched Biobank subjects that did not carry any of the tested variants were identified as controls. Variant carriers and matched controls were invited to provide an additional blood sample for plasma metabolite quantification (U and UH_2_) and PBMC‐based DPD activity measurements. The study was approved by the Mayo Clinic Institutional Review Board (protocol # 08‐00749 and 14‐005521).

### Sample processing and phenotypic assessment of DPD function

PBMCs, plasma, and genomic DNA were isolated from whole blood within 2 hours of collection using previously described methods.^9^, ^21^ PBMC DPD activity was quantified by measuring the conversion of [6‐C^14^]‐5‐FU to [6‐C^14^]‐5‐dihydrofluorouracil ([6‐C^14^]‐5‐DHFU) under standard reaction conditions in the presence of excess NADPH. Reaction products were quantified by high‐performance liquid chromatography (HPLC) as described.^9^ Plasma U and UH_2_ were quantified by liquid chromatography–tandem mass spectrometry in multiple reaction monitoring mode on a Sciex QTrap 5,500 mass spectrometer as previously described.^21^ All metabolite measures were above the previously detailed limits of detection.^21^

### Targeted high‐throughput sequencing

Subjects were previously genotyped for the *DPYD* variants c.1905+1G>A, c.1679T>G, c.2846A>T, c.1129‐5923C>G, c.85T>C (rs1801265), and c.496A>G (rs2297595) as described in.^9^, ^21^ In the present study, we performed targeted high‐throughput sequencing using Access Array chemistry (Standard BioTools, Boston, MA, USA) with target capture.^22^, ^23^ Primers were designed to amplify the exon coding regions of *DPYD* (NM_000110) and the regions surrounding c.1129‐5923C>G. Target amplification, sample indexing, and sequencing library preparation were performed using Juno Targeted Sequencing LP 192.24 Integrated Fluidic Circuits on a Juno Instrument (Standard BioTools). Pooled libraries were sequenced on an HiSeq 4000 (Illumina, San Diego, CA, USA) at the Mayo Clinic Genome Analysis Core (Rochester, MN, USA).

Demultiplexed reads underwent pruning of adapter and region‐specific primer sequences using pTrimmer.^24^ Reads were mapped to the hg19 reference genome using HiSat2.^25^ Variant calling was performed for mapped reads using the GATK germline short variant discovery pipeline, which consisted of per‐sample variant calling with HaplotypeCaller in GVCF mode, consolidation of GVCFs with GenomicsDBImport, cohort joint calling with GenotypeGVCFs, and variant filtering and recalibration with VariantRecalibrator and ApplyVSQR.^26^ Rare *DPYD* variants of interest were confirmed with Sanger sequencing using exon‐specific primers (**Table** **S1**). Population allele frequency data were retrieved from gnomAD v4.1.0^27^ exome data.

### Statistical analysis

Statistical analyses were performed in R v4.4.1 (R Foundation for Statistical Computing, Vienna, Austria) using R Studio v2023.12.1.402.1 (R‐studio Inc., Boston, MA, USA). For comparisons between groups, the base stats package in R was utilized. Comparisons for significance between two groups were performed using independent *t*‐tests in R with the t.test() function with a significance level of *P* < 0.05. Comparisons between multiple groups were performed using the aov() function in R for a one‐way ANOVA. Tukey's post hoc comparisons were performed for any ANOVA with *P* < 0.05 using TukeyHSD().

For correlation analyses among UH_2_, U, UH_2_:U, and PBMC DPD activity, data were log_10_ normalized, and linear associations were modeled using the R EnvStats package (v3.1.0). Associations between genetic variants and phenotypic measures were tested using generalized linear models incorporating prespecified covariates using the glm function. Age was defined as a continuous variable, and sex was defined as a factor. Specific models for each analysis are described in figure legends. For example, a model to test the four well‐studied *DPYD* variants (c.1905+1G>A, c.1679T>G, c.2846A>T, and c.1129‐5923C>G), sex, age, and novel/rare *DPYD* variants to determine associations of risk variants with UH_2_:U would be depicted as: glm(ratio ~ sex + age + c.1905+1G>A + c.1679T>G + c.2846A>T + c.1129‐5923C>G). Variants were considered significant with *P* < 0.05. Data were plotted using ggplot2 (v4.0.0).

## RESULTS

### Cohort characteristics and *DPYD* sequencing

The study population consisted of 203 individuals from the Mayo Clinic Biobank and was enriched for carriers of *DPYD* c.1905+1G>A, c.1679T>G, c.2846A>T, and c.1129‐5923C>G variants. The study population consisted of subjects from our previous report^9^ for which we were able to measure both PBMC DPD activity and plasma metabolite levels, and for which we had adequate DNA to perform targeted high‐throughput sequencing. Population demographics and characteristics are summarized in **Table** **1**.

**Table 1:** Population demographics and characteristics

Characteristics	All	Non‐Carrier	c.1129‐5923C>G	c.2846A>T	c.1679 T>G	c.1905+1G>A
Number of subjects	203	120	56	12	2	13
Mean age	60	60	58	55	74	65
(CV)	(0.23)	(0.21)	(0.25)	(0.27)	(0)	(0.23)
Male	83	43	28	6	0	6
(%)	(41%)	(36%)	(50%)	(50%)	(0%)	(46%)
Female	120	77	28	6	2	7
(%)	(59%)	(64%)	(50%)	(50%)	(100%)	(54%)
Mean U (ng/mL) (CV)	11.8 (0.88)	10.4 (1.08)	12.1 (0.79)	15.5 (0.35)	16.3 (0.049)	16.3 (0.47)
*P*‐value			0.79	0.037	0.0016	0.018
Mean PBMC DPD activity^a^ (CV)	217 (0.69)	254 (0.68)	177 (0.50)	177 (0.81)	81 (0.08)	118 (0.39)
*P*‐value^b^			0.0018	0.12	0.16	0.0051
Mean UH_2_:U ratio (CV)	8.7 (0.33)	9.6 (0.29)	8.4 (0.30)	5.8 (0.25)	5.1 (0.16)	5.7 (0.39)
*P*‐value^b^			0.011	1.0 × 10^−5^	0.024	4.0 × 10^−6^

^a^ Units: pmol 5‐FU min^−1^·mg^−1^.

^b^ Two‐tailed Student's *t*‐test comparing data from the indicated carrier group to the noncarrier group.

Targeted high‐throughput sequencing identified 22 variants in *DPYD* exons and adjacent intronic regions (**Table** **2**). Perfect genotype concordance with previous data was noted for the six variants that were genotyped using different approaches in our earlier studies of this cohort (c.1905+1G>A, c.1679T>G, c.2846A>T, c.1129‐5923C>G, c.496A>G, and c.85T>C). Four additional common variants (c.2194G>A, c.1896T>C, c.1627A>G, and c.1601G>A) were detected at expected frequencies based on gnomAD data. Among the remaining 12 rare variants with gnomAD allele frequencies < 1%, two (c.2193C>T and c.171T>C) were synonymous and not expected to affect protein function. Six variants (c.1781C>A, c.1278G>T, c.1218G>A, c.775A>G, c.656A>G, and c.545T>A) have previously been shown to not significantly impair DPD enzyme function *in vitro*,^28^, ^29^ and one (c.1349C>G) has been predicted to not affect enzyme activity based on *in silico* modeling.^29^

**Table 2:** *DPYD* exon variants and known deleterious variants detected in the study population

CDS position (NM_000110)	Amino acid change (NP_000101)	rsID	hg19 coordinates (chromosome 1)	Allele frequency in study	gnomAD exome allele frequency	Functional classification
c.2846A>T	p.D949V	rs67376798	97,547,947	0.030	0.0052	Decreased function^d^
c.2194G>A	p.V732I	rs1801160	97,770,920	0.033	0.047	Normal function^d^
c.2193C>T	p.G731G	rs139543981	97,770,921	0.0025	0.000085	Synonymous
c1905+1G>A	*2A	rs3918290	97,915,614	0.032	0.0051	No function^d^
c.1896T>C	p.F632F	rs17376848	97,915,624	0.042	0.047	Synonymous
c.1781C>A	p.T594N	rs759249769	97,915,739	0.0025	0.0000082	Normal function^f^
c.1679T>G	p.I560S	rs55886062	97,981,343	0.0049	0.00068	No function^d^
c.1627A>G	p.I543V	rs1801159	97,981,395	0.16	0.20	Normal function^d^
c.1601G>A	p.S534N	rs1801158	97,981,421	0.015	0.019	Normal function^d^
c.1435G>A	p.G479S	rs1651166304	98,015,205	0.0025	0.0000021	Deleterious^g^
c.1349C>G	p.A450G	rs72975710	98,015,291	0.0025	0.000018	Normal function^g^
c.1278G>T	p.M426I	rs764666241	98,039,377	0.0025	0.000028	Normal function^d^
c.1129‐5923C>G^a^	HapB3^a^	rs75017182^a^	98,039,419^a^	0.14	0.019^b^	Decreased function^d^
c.1218G>A	p.M406I	rs61622928	98,039,437	0.0025	0.0022	Normal function^d^
c.966C>A	p.C322*	rs768834079	98,058,936	0.0025	0.000031	Truncation
c.775A>G	p.K259E	rs45589337	98,144,726	0.012	0.0068	Normal function^d^
c.656A>G	p.K219R	rs781184141	98,164,931	0.0025	0.000027	Normal function^f^
c.545T>A	p.M182K	rs779728902	98,165,042	0.0025	0.000018	Normal function^f^
c.496A>G	p.M166V	rs2297595	98,165,091	0.072	0.090	Normal function^d^
c.274C>G	p.P92A	rs143986398	98,205,995	0.0025	0.00013	Decreased function^e^
c.171T>C	p.N57N	rs1452563352	98,293,732	0.0025	0.0000014	Synonymous
c.85T>C^c^	p.C29R^c^	rs1801265	98,348,885	0.30	0.22	Normal function^d^

^a^ In this study, c.1129‐5923C>G was detected in perfect linkage disequilibrium with the proxy variant c.1236G>A/rs56038477 (i.e., no individuals with evidence for recombination between these two variants were noted). Therefore, for clarity, only results for the causal variant c.1129‐5923C>G and not c.1236G>A are reported.

^b^ GnomAD exome allele frequency is reported for c.1236G>A, which is in near‐perfect LD with the intronic variant c.1129‐5923C>G.

^c^ The reference allele for rs1801265 changed between GRCh37/hg19 and GRCh38/hg38. For consistency, we are reporting the nucleotide and base changes denoted in GRCh38/hg38, which is consistent with the accepted represented base change on DPYD RefSeqGene LRG_722.

^d^ Classification as published by Clinical Pharmacogenetics Implementation Consortium (CPIC) guidelines.^10^

^e^ Classification based on *in vitro* data published in PMID: 24648345.^28^

^f^ Classification based on *in vitro* data published in PMID: 29327356.^29^

^g^ Classification based on *in silico* prediction using DPYD‐Varifier PMID: 29327356.^29^

The three remaining variants, c.274C>G, c.966C>A, and c.1435G>A, were deemed likely to be deleterious to DPD function. The missense variant c.274C>G significantly decreased DPD activity *in vitro* similar to other partially deleterious variants.^28^ The nonsense variant c.966C>A introduces a premature stop codon at DPD amino acid position 322, prior to the U/5‐FU binding domain, and is expected to completely ablate enzyme function, similar to the effect observed for the nonsense variant encoding p.E386*.^28^ The c.1435G>A missense variant encodes the amino acid change p.G479S, located near the FAD and NADPH binding domains.^28^, ^29^, ^30^ No homozygous or compound heterozygous carriers of known or suspected deleterious *DPYD* alleles were detected in this population.

### Relationship between PBMC DPD activity and plasma metabolite measures

To begin to compare approaches for identifying DPD deficiency, we evaluated the relationship between PBMC DPD activity and plasma metabolite‐based measures in matched samples. The expectation was that the enrichment for carriers of deleterious *DPYD* variants would improve the representation of individuals with low DPD activity for these analyses. A statistically significant correlation between UH_2_:U ratio and DPD activity was observed (*P* = 0.022; **Figure** **1** **a**); however, the correlation was very weak corresponding to a poor goodness of fit (*r* = 0.16; **Figure** **1** **a**). As expected, U and UH_2_ levels were strongly positively correlated (*P* < 2.2 × 10^−16^, *r* = 0.77; **Figure** **S1A**). The UH_2_:U ratio was inversely correlated with U (*P* < 2.2 × 10^−16^, *r* = −0.69; **Figure** **S1B**), while UH_2_ alone was not meaningfully correlated with the UH_2_:U ratio (*P* = 0.35, *r* = −0.066; **Figure** **S1C**). Plasma U levels were very weakly inversely correlated with PBMC DPD activity (*P* = 0.066, *r* = −0.13; **Figure** **S1D**), whereas plasma UH_2_ did not correlate with PBMC DPD activity (*P* = 0.60, *r* = 0.037; **Figure** **S1E**).

**Figure 1:**
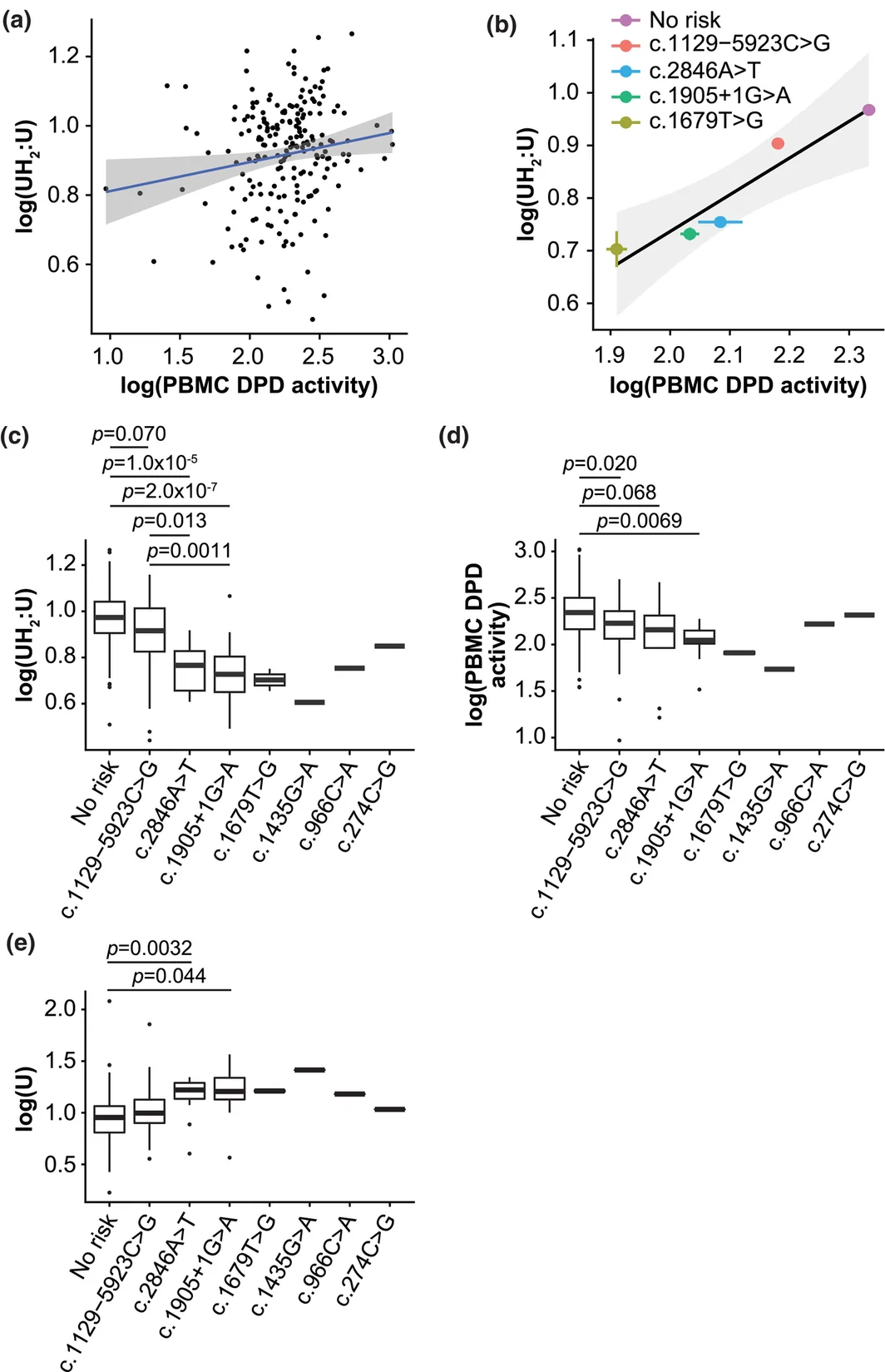
(a) The correlation between UH_2_:U and PBMC DPD activity was determined using a linear model. Trendline (blue) and 95% confidence interval (gray) are shown. (b) Mean UH_2_:U and PBMC DPD activity were summarized by genotype group. Error bars represent the standard error of the mean. Samples included in the analysis include carriers of no risk variants (*n* = 117), c.1129‐5923C>G (*n* = 56), c.2846A>T (*n* = 12), c.1905+1G>A (*n* = 13), and c.1679T>G (*n* = 2). Carriers of the rare variants c.274C>G (*n* = 1), c.966C>A (*n* = 1), c.1435G>A (*n* = 1) were not included in the analysis presented in panel b. Boxplots by genotype are presented for log_10_ normalized UH_2_:U (c) and DPD activity (d), and plasma U (e). The number of subjects carrying each genotype are the same as described for panel a. Boxes represent the first and third quartiles. Black bars indicate the median, and the whiskers represent 1.5× the interquartile range. Adjusted *P*‐values were calculated by Tukey's post hoc comparisons of means for all possible comparisons. Adjusted *P*‐values ≤ 0.1 are shown, with < 0.05 considered statistically significant.

To assess whether genotype influences these relationships, analyses were stratified by *DPYD* genotype groups. First, the group mean was plotted for noncarriers and carriers of c.1129‐5923C>G, c.2846A>T, c.1905+1G>A, and c.1679T>G (**Figure** **1** **b**). Carriers of c.1435G>A, c.966C>A, and c.274C>G were removed for this analysis (*n* = 1 for each variant). When aggregated to genotype‐group means, UH_2_:U and PBMC DPD showed a very strong linear relationship (*r* = 0.95, *P* = 0.012), with both measures capturing genotype‐associated shifts in DPD function (**Figure** **1** **b**).

We next investigated correlations within genotypes stratified by functional groupings. Because of similarity in function and clinical risk, the two carriers of c.1679T>G were grouped with carriers of c.1905+1G>A. Carriers of rare variants that are potentially deleterious (i.e., c.1435G>A, c.966C>A, and c.274C>G) were plotted as individual points and not included in correlation calculations. Overall, correlations between measures within genotypes (**Figure** **S2**) were similar to those observed on the overall dataset (**Figures** **1a** **and** **S1A–E**), and no significant correlations were observed between PBMC DPD activity and UH_2_:U ratio within any individual genotype group (**Figure** **S2A**; *P* > 0.3 for all comparisons).

### Association of *DPYD* variants with measures of DPD function

We next tested whether phenotypic measures differed across genotype groups. When comparing the UH_2_:U ratio among genotype groups, there is a significant overall difference by genotype (*p*_*Anova*_ = 2.0 × 10^−11^; **Figure** **1** **c**). Tukey *post hoc* comparisons indicated a significantly decreased UH_2_:U ratio in c.1905+1A and c.2846T carriers in comparison to subjects who carried no risk variants (*P* = 2.0 × 10^−7^ and *P* = 1.0 × 10^−5^, respectively). Additionally, UH_2_:U ratios in c.1905+1A and c.2846T carriers were significantly lower than those in c.1129‐5923G carriers (*P* = 0.0011 and *P* = 0.013, respectively). UH_2_:U ratios also trended lower in carriers of c.1129‐5923C>G compared with carriers of no risk variants; however, the difference was not significant (*P* = 0.070). Decreased UH_2_:U ratios were also observed for c.1679G and c.1435A carriers, although statistical inference was limited by small sample sizes (*n* = 2 and *n* = 1, *P* = 0.11 and *P* = 0.12, respectively).

PBMC DPD activity similarly differed across genotype groups (*p*_*Anova*_ = 4.1 × 10^−5^; **Figure** **1** **d**) with significantly reduced PBMC DPD activity observed in carriers of c.1129‐5923G and c.1905+1A measures compared with the no risk variant group (*P* = 0.020, *P* = 0.0069, respectively; **Figure** **1** **d**). PBMC DPD also trended lower in c.2846A>T carriers, but the difference was not significant (*P* = 0.068).

When assessing individual metabolite measures, U levels also showed an overall significant difference between genotype groups (*p*_*Anova*_ = 2.2 × 10^−4^). Significantly increased U was noted in c.1905 + 1A carriers relative to the no risk group (*P* = 0.0032; **Figure** **1** **e**) and in c.2846T carriers in comparison to the no risk group (*P* = 0.044; **Figure** **S1E**). No differences in UH_2_ across genotype groups were observed (*p*_*Anova*_ = 0.99; **Figure** **S3**).

Next, we performed multivariable linear regression modeling that included sex, age, and *DPYD* variant status. Genetic predictors included the four well‐studied deleterious variants (i.e., c.1905+1G>A, c.1679T>G, c.2846A>T, and c.1129‐5923C>G), the comparatively common variant c.496A>G (previously linked to reduced DPD function,^21^, ^31^) and a pooled indicator for rare variants that were deemed to be likely deleterious (i.e., c.1435G>A, c.966C>A, and c.274C>G). Six of 29 carriers of c.496A>G also carried other variants included in the linear model—four carried c.1129‐5923C>G, one carried c.2846A>T, and one carried c.1905+1G>A. All other variants were present as single heterozygous alleles.

As expected, the four well‐studied deleterious variants were significantly associated with reduced UH_2_:U ratios (**Figure** **2** **a**). The effect of c.1129‐5923C>G on UH_2_:U was comparatively modest (−16.5%), whereas c.2846A>T (−40.9%), c.1905+1G>A (−43.0%), and c.1679T>G (−46.5%) showed larger reductions. c.496A>G was also independently associated with a 19.1% reduction in UH_2_:U ratio (*P* = 0.00070), which is similar to the 16.5% reduction observed for c.1129‐5923G>A (**Figure** **2** **a**). When considered as a group, the three rare variants were associated with a 44.0% reduction UH_2_:U ratio (*P* = 0.0012), comparable to that of the established risk‐alleles c.2846A>T, c.1905+1G>A, and c.1679T>G (**Figure** **2** **a**). A secondary model treating these three rare variants as individual factors yielded similar results (**Figure** **S4A**).

**Figure 2:**
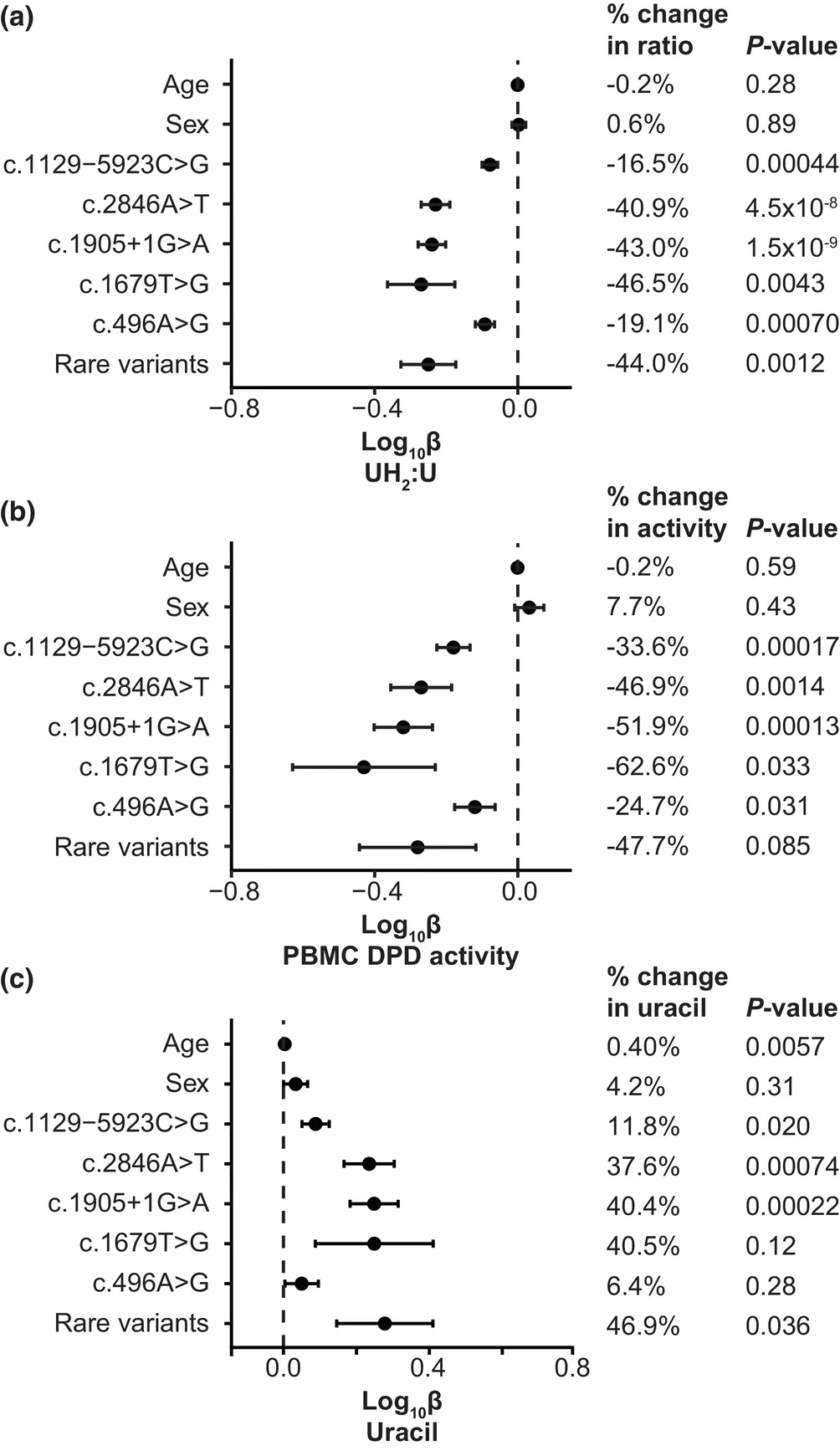
Associations between *DPYD* variants and UH_2_:U ratio (a), PBMC DPD activity (b), and U levels (c) are depicted. Linear modeling was performed in R using the glm function with the modeling represented as glm(Phenotypic measure ~ Sex + Age + each clinically actionable *DPYD* variant + Rare). The reference group for sex is female, with changes in phenotypic measure representing the changes for males. The rare variant group (*n* = 3) consists of c.274C>G, c.966C>A, and c.1435G>A. For all measures, the sample sizes are: no risk group (*n* = 117), c.1129‐5923G carriers (*n* = 56), c.2846T carriers (*n* = 12), c.1905+1A carriers (*n* = 13), and c.1679G carriers (*n* = 2). Error bars depict the standard error. Variants significantly associated with a decreased UH_2_:U are defined by *P* < 0.05.

Linear regression modeling of PBMC DPD activity produced similar results, with c.1129‐5923C>G associated with a modest 33.6% reduction in activity compared with the other well‐studied variants (range −46.9% to −62.6%; **Figure** **2b**). Likewise, c.496A>G was associated with a 24.7% reduction in activity (*P* = 0.031). Pooled rare variants showed a 47.7% reduction in activity but failed to reach significance (*P* = 0.085). In the secondary linear model where the rare variants were treated independently, c.1435G>A was again associated with a more severe decrease in DPD activity (**Figure** **S4B**), similar to the effect noted in analyses of UH_2_:U. Across all models tested, age and sex did not significantly contribute (**Figures** **2** **and** **S4**).

The same modeling was performed with U as an endpoint. Three variants (c.1129‐5923G, c.2846T, and c.1905+1A) were associated with significantly increased U levels, as was the collective group of three rare deleterious *DPYD* variants, consistent with DPD deficiency (**Figure** **2** **c**). However, significantly increased U levels were not associated with c.496G or c.1679G (**Figure** **2** **c**). When treating rare variants as independent factors, c.1435A showed a large increase in U (158.5%) compared with other tested variants (**Figure** **S4C**). Interestingly, while sex was not significant, age was associated with a significant increase in U (0.40%, *P* = 0.0057; **Figure** **2** **c**).

### Sex‐stratified differences in pyrimidine catabolism

5‐FU toxicity to standard doses is more prevalent in females than males^32^, ^33^, ^34^; however, in linear models, sex did not significantly contribute to differences in DPD function as a covariable (**Figures** **2** **and** **S4**). To investigate sex as a biological variable in greater detail, we directly compared metabolite and PBMC DPD activity measurements between sexes within genotype groups.

Among carriers of nonfunctional *DPYD* variants, males exhibited lower UH_2_:U ratios than females (*P* = 0.020; **Figure** **3a**). No significant differences between sexes were observed in UH_2_:U data within other genotype groups (all *P* > 0.10; **Figure** **3** **a**). A trend toward decreased DPD activity within female nonfunctional variant carriers in comparison to males was noted within the same genotype group (*P* = 0.079), whereas no significant differences were apparent among variant carrier groups (all *P* > 0.10; **Figure** **3** **b**). Plasma U was significantly higher in males than females within the nonfunctional variant group (*P* = 0.024; **Figure** **3** **c**). Within the noncarrier (no risk) group, we observed a slight trend toward higher U in males compared with females (*P* = 0.14); however, no significant differences in any group except carriers of nonfunctional variants (all other *P* > 0.10; **Figure** **3** **c**). Plasma UH_2_ was significantly higher in males than females within the noncarrier group (*P* = 0.032), whereas no sex differences were observed in any of the variant carrier groups (all *P* > 0.27; **Figure** **3** **d**).

**Figure 3:**
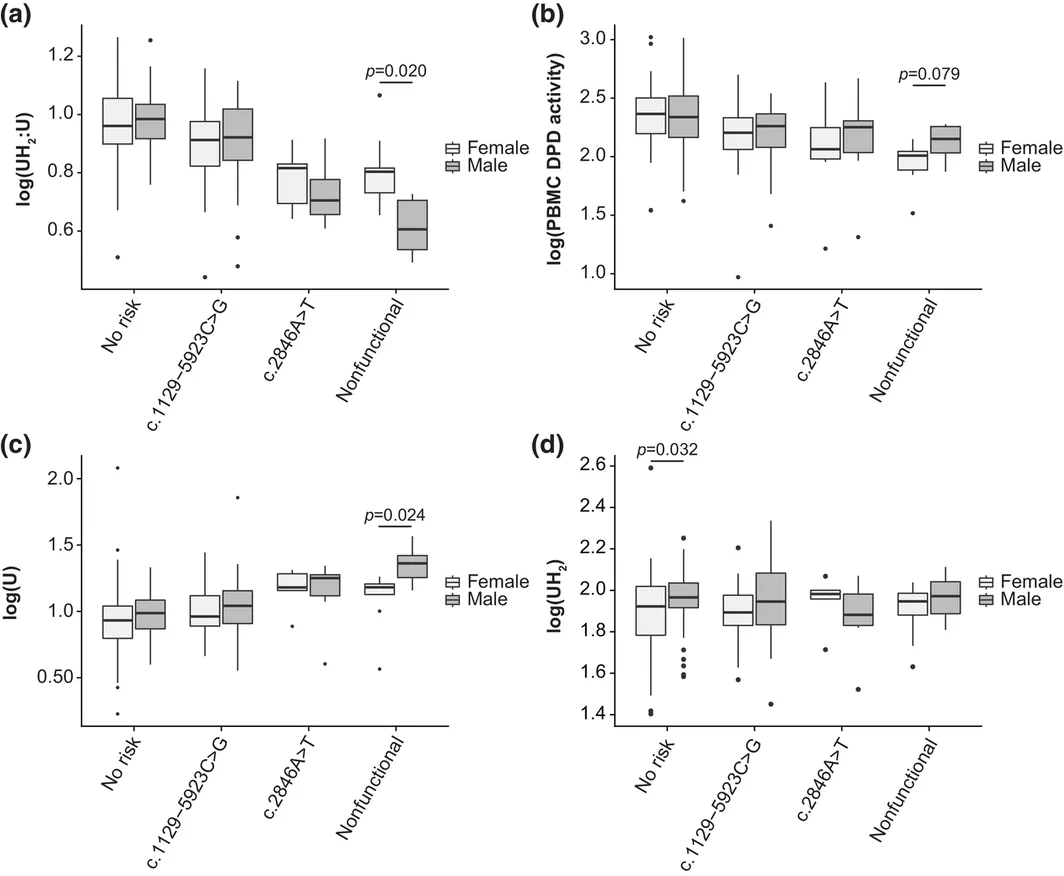
Measures of DPD activity, including plasma UH_2_:U ratio (a), PBMC DPD activity (b), plasma U (c), and plasma UH_2_ (d) were compared between sexes within genotype groups. For each panel, there were 41 male and 76 female carriers of no risk variants, 28 male and 27 female carriers of c.1129‐5923G, 6 male and 6 female carriers of c.2846T, and 6 male and 9 female carriers of nonfunctional variants, which included c.1905+1A and c.1679G. Three carriers of the rare variants c.274G, c.966A and c.1435A were not included in these analyses. Boxes represent the first and third quartile. Bars represent medians, and whiskers represent 1.5× the interquartile range. Student's *t*‐tests were used for pairwise comparisons of values between male and female carriers of each genotype group. *P*‐values ≤ 0.1 are shown, with < 0.05 considered statistically significant.

### Using phenotypic measures of DPD activity to guide 5‐FU dosing

While specific UH_2_:U and PBMC DPD activity thresholds for assigning DPD deficiency have not been unanimously defined, < 70% of the mean value observed in individuals who do not carry a risk variant for 5‐FU‐related toxicity has been suggested as predictive of clinically relevant DPD deficiency and elevated 5‐FU toxicity risk.^35^ We derived this value from our data (6.76 for UH_2_:U and 180 for PBMC DPD activity), which is similar to the threshold of 6 used for UH_2_:U in a previous study^36^ and identified the proportion of subjects with known deleterious *DPYD* genetic variation that would be successfully identified using this approach. For plasma U, > 16 ng/mL was used as the threshold for DPD deficiency, in line with much of the literature and guidelines from the European Medicines Agency (EMA).^15^, ^37^, ^38^

Among individuals not carrying any deleterious *DPYD* variant, ~ 14% were DPD‐deficient based on UH_2_:U (**Figure** **4** **a**), compared with ~ 35% based on DPD activity (**Figure** **4** **b**). For plasma U, < 7% of noncarriers were above the threshold of 16 ng/mL (**Figure** **4** **c**). When carriers of deleterious *DPYD* variants are assessed, ~ 85% of c.1905+1A carriers, along with all c.1679T>G, c.966C>A, and c.1435G>A carriers, are captured as DPD deficiencies using UH_2_:U ratios and PBMC DPD activity (**Figure** **4** **a,b**, respectively). DPD activity identified 54% of c.1129‐5923C>G carriers as DPD‐deficient, whereas 29% of carriers were captured using UH_2_:U. On the other hand, the UH_2_:U ratio captures more carriers of c.2846A>T below the threshold (75%) in comparison to DPD activity, which only captures 58% of individuals below the threshold (**Figure** **4** **a**). In comparison, when a threshold plasma U concentration of 16 ng/mL was used, fewer variant carriers, as a whole, were deemed DPD‐deficient—12.5% of c.1129‐5923C>G, 50% of c.2846A>T, 54% of c.1905+1G>A, and 50% of c.1679T>G (**Figure** **4** **c**).

**Figure 4:**
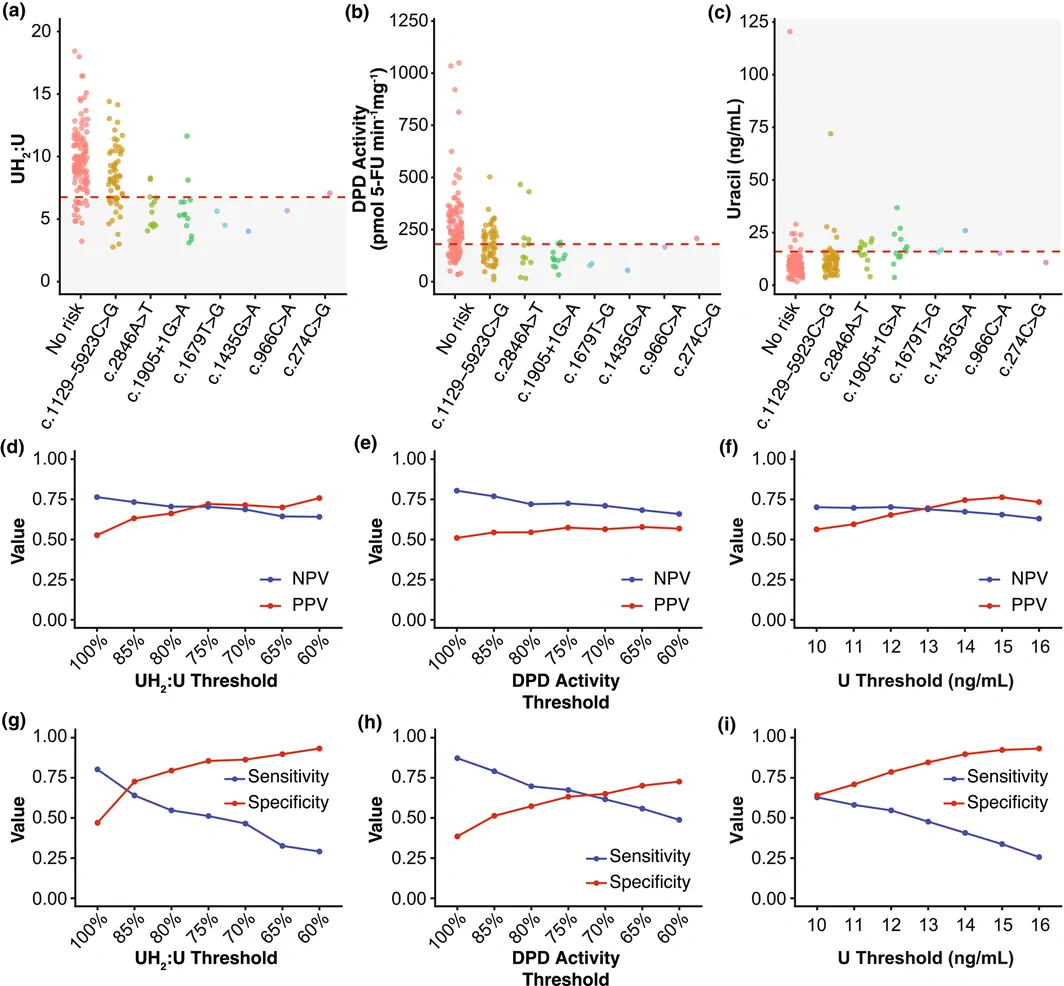
Toxicity threshold modeling was performed using UH_2_:U ratio, PBMC DPD activity, and plasma U levels. Thresholds for assigning a functional classification of DPD deficiency were defined as < 70% of the mean plasma UH_2_:U ratio (a) or PBMC DPD activity (b) measured in individuals that did not carry a known or predicted *DPYD* allele associated with impaired DPD function (i.e., the “no risk” group). For plasma U, a threshold of > 16 ng/mL was used. The number of subjects in each genotype group was as described for **Figure** **1** **b**. Negative predictive value (NPV) and positive predictive value (PPV) for plasma UH_2_:U ratio (d), PBMC DPD activity (e), and plasma U were plotted across varying thresholds. Sensitivity and specificity for plasma UH_2_:U ratio (e), PBMC DPD activity (f), and plasma U levels were calculated using varying thresholds. Thresholds for UH_2_:U are defined as follows: 100% (9.66), 85% (8.21), 80% (7.73), 75% (7.24), 70% (6.76), 65% (6.28), and 60% (5.79). For PBMC DPD activity, the thresholds are: 100% (257.15), 85% (218.57), 80% (205.72), 75% (192.86), 70% (180.00), 65% (167.15), and 60% (154.29).

Positive predictive value (PPV), negative predictive value (NPV), sensitivity, and specificity were calculated for threshold ranges of 60–100% of the noncarrier average (UH_2_:U and PBMC DPD activity) and 10–16 ng/mL (U). As expected, PPVs trended upwards and NPVs trended downwards as more stringent thresholds were applied across all methods of assessing DPD function (**Figure** **4** **d–f**). It is noted that the subject selection strategy enriched for carriers of deleterious variants, which would impact PPV and NPV estimates. Sensitivity and specificity should not be affected, however. Both UH_2_:U and U showed higher specificity compared to PBMC DPD activity across the ranges evaluated, although sensitivity was reduced (**Figure** **4** **g–i**).

## DISCUSSION

In this study, we directly compared three commonly used measures of systemic DPD activity, plasma UH_2_:U, plasma U, and PBMC DPD activity. While all measures show significant differences between carriers of severely deleterious *DPYD* variants and noncarriers (**Figure** **2**), PBMC DPD activity poorly correlated with metabolite measures and ratios (**Figure** **1** **a**) which is consistent with previous studies^39^ and calls into question whether PBMC DPD activity should be considered the “gold standard” functional approach for identifying DPD‐deficient individuals. When comparing DPD activity measures by genotype, UH_2_:U showed an improved ability to differentiate carriers of the partially deleterious variants c.1129‐5923C>G and c.2846A>T from subjects who did not carry a deleterious *DPYD* variant (**Figure** **1** **c,d**), although appreciable overlap is noted between data distributions by genotype. Interestingly, the ability to identify significant differences in DPD activity by genotype was also diminished for plasma U compared to UH_2_:U (**Figure** **1** **e**).

The use of phenotypic measures of DPD function as biomarkers for DPD deficiency is appealing because they have the potential to capture cases of DPD deficiency beyond those identified by targeted genotype panels. Current guidance from the US Food and Drug Administration (FDA) and the European Medicines Agency (EMA) recommend testing for DPD deficiency prior to administering 5‐FU.^38^, ^40^ While the FDA label specifies testing for deleterious genetic variants of *DPYD*,^40^ the EMA recommends genetic and/or phenotypic testing of plasma U, with threshold values for plasma U defined as > 16 ng/mL.^38^ Using > 16 ng/mL as a stratification threshold for U, we observed reduced sensitivity for identifying individuals who carried deleterious *DPYD* variants compared with the other two phenotypic tests (**Figure** **4**). However, it is noted that in our original study, blood tubes were not placed on ice immediately upon collection, with the intent to improve PBMC recovery, potentially affecting metabolite measurements.^15^ Consequently, the variability observed in U and/or UH_2_:U measurements may be greater than would be expected under fully standardized pre‐analytical conditions. In our study, balanced specificity and sensitivity were observed when a threshold of 10 ng/mL U was used (**Figure** **4** **i**), consistent with the findings of Launay *et al*.^36^ where balanced accuracy for discriminating deleterious genotypes was noted at a threshold of 11 ng/mL.

In the Alpe2U study, administering a reduced 5‐FU dose to patients with pretreatment plasma U levels > 16 ng/mL significantly reduced severe toxicity.^41^ However, 5‐FU exposure was also markedly reduced in nonvariant carriers with U > 16 ng/mL compared with controls.^41^ This issue would be expected to be exacerbated if a relaxed U threshold were applied (i.e., more false‐positives would likely be identified). It is also noted that high variability and poor correlations were observed for all phenotypic measures across genotypes (**Figures** **1a** **and** **S1D**) and within carriers of specific genotypes (**Figure** **S2A,E**) in the present study. Collectively, these findings suggest that phenotypic measures may have limited diagnostic value at the individual level but may retain utility for identifying rare cases of profound DPD deficiency of unknown cause^42^ and for classifying variants when population‐level data from multiple carriers are available.^43^ Further study is needed to identify trade‐offs between toxicity and drug exposure, as well as metrics of efficacy, when initial dose adjustment is performed on phenotypic or genotypic biomarkers of DPD deficiency.

Within this subject cohort, we identified three ultrarare variants associated with reduced UH_2_:U ratios (c.274C>G, c.966C>A, and c.1435G>A). In previous studies, we showed that the amino acid change encoded by c.274C>G led to significantly reduced DPD activity *in vitro*.^28^ The c.966C>A variant introduces a premature stop codon at amino acid 322, resulting in a severely truncated protein. Functional characterization of other truncation variants, including a truncation at amino acid 386, has demonstrated a complete loss of DPD function, likely due to the lack of critical domains necessary for enzyme function.^28^ To the best of our knowledge, functional data for c.1435G>A have not yet been reported; however, *in silico* prediction using a gene‐specific model suggests that the encoded amino acid change (p.G479S) is likely deleterious to DPD function.^29^ Amino acid 479 is located proximal to FAD and NADPH binding domains,^28^, ^29^, ^30^ and mutation of this residue likely disrupts electron transport mechanisms required for DPD activity.

Previous studies have suggested that c.496A>G, encoding p.M166V, contributes to reduced DPD function in a haplotype‐dependent manner.^21^ Specifically, linkage disequilibrium (LD) between c.496A>G and c.1129‐5923C>G may influence the effect size attributed to either variant in single‐marker analyses. Additionally, both c.496A>G and c.1129‐5923C>G are in LD with the c.85T>C in this cohort, a variant that has been linked to increased DPD activity.^21^ Significantly reduced UH_2_:U ratios were observed in individuals carrying c.496A>G compared to controls in this analysis, supporting a partially deleterious role for this variant based on both plasma UH_2_:U (**Figures** **2a** **and** **S4A**) and PBMC DPD activity measurements (**Figures** **2b** **and** **S4B**).

Females treated with 5‐FU experience more extensive toxicity reflected by higher maximum toxicity grades, a broader range of toxicity symptoms, and a higher overall incidence of toxicity.^1^, ^32^, ^34^, ^44^, ^45^ In one study, severe toxicity occurred in 18% of females compared with 9% of males.^46^ Dose‐limiting toxicity from the 5‐FU prodrug capecitabine has also been reported more frequently in female colorectal cancer patients.^47^ We postulate that these differences may partly reflect average differences in the metabolic compartment size between males and females. In our study, comparisons of metabolite levels between males and females without deleterious *DPYD* genetic variation revealed significantly lower plasma UH_2_ levels in plasma from female subjects (**Figure** **3** **d**). Notably, plasma U levels were similarly decreased (**Figure** **3** **c**), explaining the apparent lack of difference in UH_2_:U ratios between sexes (**Figure** **3** **a**). These findings are consistent with reports of reduced clearance of both 5‐FU and 5‐FUH_2_ in female patients, resulting in elevated 5‐FU exposure.^48^, ^49^, ^50^ It is noted that for carriers of either c.1905+1G>A and c.1679G, UH_2_:U ratios were elevated and U levels decreased in females (*n* = 8) compared with males (*n* = 7; **Figure** **3** **a**). Given the small sample size for carriers of these variants, clear conclusions cannot be drawn, however. Overall, these findings suggest that current BSA‐based dosing strategies may not adequately address sex‐specific differences in 5‐FU pharmacokinetics.

In conclusion, although plasma metabolite measures were superior at stratifying deleterious *DPYD* variant carriers compared with PBMC DPD activity, substantial intra‐genotype variability was noted across all measures. These findings support the use of phenotypic biomarkers as complementary tools rather than standalone diagnostic tests, reinforcing the central role of genetic testing in pretreatment risk stratification for fluoropyrimidine therapy.

## FUNDING

This work was supported by the National Cancer Institute of the National Institutes of Health under award number R01CA251065 (SMO, PI), the Swiss National Science Foundation under award number 320030_212583 (CRL, PI), and the National Institute of Neurological Disorders and Stroke of the National Institutes of Health under award number 1F31NS141217 (BMB, PI). The funding organizations did not contribute to the study design, the collection, analysis, and interpretation of data, or in writing the manuscript.

## CONFLICT OF INTEREST

The authors declared no competing interests for this work.

## AUTHOR CONTRIBUTIONS

B.M.B, S.H., K.J.B., C.R.L., and S.M.O. wrote the manuscript; C.R.L and S.M.O. designed the research; B.M.B., S.H., and K.J.B. performed the research; B.M.B. analyzed the data.

## Supporting information


Data S1.
