# Supplementary material for: Dihydropyrimidine Dehydrogenase Genotype–Phenotype Correlations in Plasma and Peripheral Blood Mononuclear Cells
Source: Clin Pharmacol Ther. 2026 Oct 6:10.1002/cpt.70511. Online ahead of print. doi: 10.1002/cpt.70511 (PMC13643120; doi:10.1002/cpt.70511)
Supplement: Supplementary file 1 [file CPT-9999-0-s001.docx]

**Supplementary Tables**

| **Table S1. Amplification and sequencing primers used to confirm genotype status of rare variants.** | | |
| --- | --- | --- |
| ***DPYD* Exon** | **Forward Primer 5’-3’** | **Reverse Primer 5’-3’** |
| Exon 2 | CTGGAAGGCAGAGGTTGCAGTG | AAGCAGTGTAATTAAAAATACACTTTCAAACTTT |
| Exon 6 | GTGTTTCCTAGAGGAGGCATGACTC | TCTGTGAGCCTGAAGTTCCTATATGA |
| Exon 9 | CCCTGTAATGTTATATATGTTTATGTGTGT | ATTCCTTACGATGATACTTTATTGGAAG |
| Exon 10 | TTCATCCTGGAAAACTGCAAGATGC | TCTGAATTAGAAAAGAAACAATTATGTGTAGCC |
| Exon 11 | ACAAAATGCAAATAACTGGTAACTGAAACT | CATTTCTAAGCCTTTTGAATCAAGATTGC |
| Exon 12 | TCCAATATTTCGTTAGCTTTTCATTTTTAT | ACTCAACAGTTCTACTACCTAGTCTAC |
| Exon 13 | TTCATACTGCCTTTGAAATTAAAAGGCCAT | TTGTTGCTTCCTAGACAATACTAAATGAGCAATA |
| Exon 14 | AAAATTCCTCTGCAAAAATGTGAGAAGGG | ATAACAAAGCTTCACATTGTGTGGGTTTT |
| Exon 15 | TCACGTACATGCATGTATTTACATTGCTACT | ACATTTCCATCATTTTAATGAAAACACAGGGT |
| Exon 16 | GCACTGAGTAAATATTTGGTGTGTGATTGT | AGTTGCTATAATTTCAGCTTCCCTCATTTTC |
| Exon 17 | TTTCTAAAGGCTCTGTTGAGGAATTTAACCT | AGGAAAAACTATAAAGGATCTTGTGTTTCCAG |
| Exon 19 | CTACTTGTATAACATGTCTAAATTCAATATTGG | GAAAAGACAGTGGGTTCGTAAGCCC |
| Exon 20 | ACCATTACCATTGCAAGTTTATGAAAAATGT | TGGTTTATAAAAAGGTAACTCACAGACCCA |
| Exon 22 | AATTAGGTCTCGTATTTCTAAGCCAGGC | ATTCTGTGCATTAAATGCTGGCATAGTTAG |
| Exon 23 | TGACATATTTTCCTCACACAATGAAGAAA | ATCAAATATGGAGCACAGCATAGGG |

**Supplementary Figures**

**Figure S1**. Correlations between each metabolite and phenotypic measures of DPD function. The correlation between each metabolite or activity measure was determined using a linear model, and the trendline represents a 95% confidence interval. **(A)** Correlation between U (ng/mL) and UH_2_ (ng/mL); R^2^ = 0.60, *p* < 2.2x10^-16^. **(B)** Correlation between U (ng/mL) and the UH_2_:U ratio; R^2^ = 0.47, *p* < 2.2x10^-16^. **(C)** Correlation between UH_2_ and the UH_2_:U ratio; R^2^ = 4.4x10^-3^, *p* = 0.35. **(D)** Correlation between U (ng/mL) and DPD activity; R^2^ = 0.017, *p* = 0.066. **(E)** Correlation between UH_2_ (ng/mL) and DPD activity; R^2^ = 1.4x10^-3^, *p* = 0.60.

**Figure S2**. Correlations between plasma metabolite measures and PBMC DPD activity within genotype groups using linear models. Sample sizes for all comparisons: no risk group (n = 117), c.1129-5923G carriers (n = 56), c.2846T carriers (n = 12), and nonfunctional variant carriers (n = 15). The nonfunctional variant group includes c.1905+1A (n = 13) and c.1679G carriers (n = 2). Correlations were not calculated for variants with a single carrier (i.e., c.274G, c.966A, and c.1435A). Gray represents the 95% confidence intervals. **(A)** Correlations between PBMC DPD activity and UH_2_:U ratios: no risk (R^2^ = 7.9x10^-4^, *p* = 0.76), c.1129-5923G (R^2^ = 4.1x10^-3^, *p* = 0.64), c.2846T (R^2^ = 0.084, *p* = 0.36), nonfunctional (R^2^ = 0.079, *p* = 0.31). **(B)** Correlations between U and UH_2_: no risk (R^2^ = 0.71, *p* < 2.2x10^-16^), c.1129-5923G (R^2^ = 0.56, *p* = 4.5x10^-11^), c.2846T (R^2^ = 0.76, *p* = 2.1x10^-4^), and nonfunctional (R^2^ = 0.62, *p* = 5.4x10^-4^). **(C)** Correlations between U and the UH_2_:U: (R^2^ = 0.33, *p* = 1.1x10^-11^), c.1129-5923G (R^2^ = 0.44, *p* = 2.7x10^-8^), c.2846T (R^2^ = 0.48, *p* = 0.013), and nonfunctional (R^2^ = 0.71, *p* = 7.7x10^-5^). **(D)** Correlations between UH_2_ and the UH_2_:U: (R^2^ = 1.8x10^-3^, *p* = 0.65), c.1129-5923G (R^2^ = 4.0x10^-5^, *p* = 0.96), c.2846T (R^2^ = 0.061, *p* = 0.44), and nonfunctional (R^2^ = 0.11, *p* = 0.23). **(E, F)** Correlations between U or UH_2_ compared to PBMC DPD activity: no risk (R^2^ = 8.7x10^-5^, *p* = 0.92 and R^2^ = 5.1x10^-5^, *p* = 0.94), c.1129-5923G (R^2^ = 0.011, *p* = 0.44 and R^2^ = 7.0x10^-3^, *p* = 0.54), c.2846T (R^2^ = 0.28, *p* = 0.076 and R^2^ = 0.26, *p* = 0.087), and nonfunctional (R^2^ = 0.066, *p* = 0.36 and R^2^ = 0.016, *p* = 0.65). Statistics are listed for U vs PBMC DPD activity and UH_2_ vs PBMC DPD activity, respectively.

**Figure S3.** Boxplots comparing plasma UH_2_ between genotypes. From left to right, the groups carry: no risk variants (n = 117), c.1129-5923G (n = 56), c.2846T (n = 12), c.1905+1A (n = 13), c.1679G (n = 2), c.966A (n = 1), and c.1435A (n = 1), c.274C>G (n = 1). All *DPYD* risk carriers were heterozygous and carried a single indicated variant. Boxes represent the first and third quartiles. Black bars represent the median, and whiskers represent 1.5x the interquartile range. Boxes represent the first and third quartiles. Black bars indicate the median, and the whiskers represent 1.5x the interquartile range. Adjusted p values were calculated by Tukey post-hoc comparisons of means for all possible comparisons. No significant differences (*p*<0.05) were noted.

**Figure S4**. Associations between *DPYD* variants and UH_2_:U ratio **(A)**, PBMC DPD activity **(B)**, or U **(C)** were assessed using linear modeling in R using the glm function with the modeling represented as glm(Phenotypic measure ~ Sex + Age + each clinically actionable *DPYD* variant + rare variants). The reference group for sex is female, with changes in phenotypic measure representing the changes for males. Each rare variant was treated as an individual variable: c.274C>G (n = 1), c.966C>A (n = 1), and c.1435G>A (n = 1). For all measures, the sample sizes are: no risk (n = 117), c.1129-5923G carriers (n = 56), c.2846T carriers (n = 12), c.1905+1A carriers (n = 13), and c.1679G carriers (n = 2). Each point represents the log_10_β for each defined variable, with error bars representing the standard error. *P* values <0.05 are considered significant.
